# Impact of biological maturation on match workload in Portuguese elite male academy soccer players

**DOI:** 10.5114/biolsport.2026.161102

**Published:** 2026-04-20

**Authors:** Nuno Ribeiro, Oliver Gonzalo-Skok, João Valente-dos-Santos, Mauro Mandorino, Marco Beato, Jamie Salter, Hadi Nobari, Nuno Loureiro, Ruben Ferreira, João Pedro Araújo, Francisco Tavares

**Affiliations:** 1 Faculty of Physical Education and Sport, Universidade Lusófona, 1749-024 Lisbon, Portugal; 2 CIDEFES, Research Center in Sports, Physical Education, Exercise and Health, Universidade Lusófona, 1749-024 Lisbon, Portugal; 3 Medical and Performance Department, Sporting Clube de Portugal, Lisbon, Portugal; 4 Department of Communication and Education, Universidad Loyola Andalucía, Seville, Spain; 5 CIFI2D, Centre of Research, Education, Innovation and Intervention in Sport, University of Porto, Porto, Portugal; 6 COD, Center of Sports Optimization, Sporting Clube de Portugal, 1600-464 Lisbon, Portugal; 7 Performance and Analytics Department, Parma Calcio 1913, 43121 Parma, Italy; 8 Department of Movement, Human and Health Sciences, University of Rome “Foro Italico”, Piazza L. de Bosis 6, 00135 Rome, Italy; 9 School of Health and Sports Sciences, University of Suffolk, Ipswich IP4 1QJ, United Kingdom; 10 School of Science, Technology and Health, York St John University, York, United Kingdom; 11 Department of Exercise Physiology, Faculty of Educational Sciences and Psychology, University of Mohaghegh Ardabili, Ardabil 56199-11367, Iran; 12 Faculty of Sport Sciences, University of Extremadura, 10003 Cáceres, Spain

**Keywords:** Adolescent Performance, External Load, Match Demands, Somatic Maturity, Youth Soccer

## Abstract

Youth soccer academies are essential for identifying and developing talented players, yet substantial inter-individual variability in biological maturation may influence match workload beyond chronological age. This study investigated the independent and interactive effects of biological maturation and age on match workload in elite male youth soccer players (U14–U17). Sixty Portuguese academy players were assessed at six time points across two competitive seasons (2021–22 and 2022–23). Biological maturation was classified using the percentage of predicted adult height, grouping players as pre-/circa-PHV and post-PHV. Mixed-effects models were used to examine the effects of age, maturity status, and their interaction on match workload metrics. Chronological age significantly influenced high-speed running (β = 0.527, p = 0.001) and sprint distance per minute (β = 0.174, p = 0.001). In contrast, total distance per minute and total accelerations and decelerations per minute were shaped by significant age × maturity interactions (p ≤ .001), indicating stronger maturityrelated effects at younger ages that attenuated across adolescence. Chronological age alone does not adequately account for variability in match workload among elite youth soccer players. Biological maturation plays a significant role, in conjunction with age, in determining the demands of total distance and acceleration/deceleration, with more pronounced effects observed at younger ages and a diminishing influence as players reach post-peak height velocity (PHV) stages. Conversely, high-speed running and sprinting distances are primarily influenced by age. These findings underscore the necessity of incorporating biological maturity into the interpretation of match workload, particularly during early to-mid adolescence.

## INTRODUCTION

Soccer academies aim to identify and develop young, talented players who can thrive at higher levels and potentially earn professional contracts [1–3]. Research on youth soccer players in academies shows that 60–80% of U16 and U17 athletes have a bone age exceeding their actual age by at least one year [4, 5]. Variations in biological maturation complicate the identification of players likely to excel at the senior level, affecting selection, physical ability, and performance [6]. From the beginning of puberty, boys who develop earlier than their peers have a significant advantage in terms of size and athletic ability [7, 8]. Extensive research has established the influence of biological maturation on the physical performance of young athletes, highlighting distinct advantages for early maturing individuals in terms of strength, speed, and power [9–13]. These players outperform their peers in speed, strength, power, agility, and endurance tests [11, 13]. Despite age-based grouping in academies, there are significant physical differences. Children of the same age can differ in skeletal age by up to five years, indicating different maturation levels [13]. Advancedaged male players exhibit greater height, weight, and mass-for-stature than their peers [11, 13, 14]. These differences include approximately 50% of body mass, 29 cm in height, 10–15% in the percentage of predicted adult height (PPAH), and 3–8.6 kg in fat-free mass [15, 16]. Maturation-related factors are likely to influence performance [17]. Thus, significant physical developmental differences among pubertal athletes complicate talent identification [18].

Recent studies have also linked training load and maturation status with muscle soreness, fatigue, and workload demands in youth soccer players [19–22]. Most research on workload relationships has focused on adult populations, with limited studies targeting younger athletes (i.e., < U-16) [23–25]. Adolescents are particularly vulnerable because of their rapid growth and maturation, and inappropriate workloads may adversely affect their development and risk of injury [26, 27]. Some studies have consistently demonstrated that biological maturation is positively correlated with superior physical performance in young soccer players. Buchheit et al. [28] found that earlier-maturing players, aged 13–18, outperformed later-maturing players in key physical metrics such as maximum speed, high-speed distance covered, and overall intensity during competitive play. This pattern contributes to the overrepresentation of early maturing players in soccer academies because late-maturing players face athletic disadvantages during competitions, which could hinder their progression to higher levels of competition. Buchheit et al. [17] confirmed these findings in U15 players, showing that those advanced in maturation reached higher peak speeds and covered greater distances at speeds above 16 km/h, mainly in midfielders and wingers. Interestingly, no significant differences in the total distance covered were observed between early- and late-maturing players, suggesting that match running metrics may depend more on playing position than on overall physical capacity.

There are several methods for estimating biological maturity [29]. Multiple approaches exist for evaluating an individual’s biological maturation, as this process affects various systems (including somatic, skeletal, endocrine, sexual, and dental systems). Researchers have previously utilized multiple indicators to assess biological maturity in young soccer players, including secondary sexual characteristics, bone age, chronological age at which peak height velocity (PHV) occurs, and testicular size [1, 2, 30–34]. Among these methods, skeletal age is typically considered the most objective and clinical technique for determining biological maturity. Nevertheless, its application in academy soccer is often restricted because of the invasive nature of the procedure, the requirement for radiation exposure, and related ethical issues [35, 36]. Previous studies investigating the relationship between biological maturation and match-running metrics have used methods such as the Mirwald maturity offset [37] or maturity ratio [38]. However, these methods have limitations, as they may not fully capture the intricacies of biological development. A major limitation of the Mirwald method is that it tends to overestimate or underestimate the age at PHV, depending on whether the athlete is early or late maturing, particularly in individuals who are far from their predicted PHV [36, 39]. This limits their precision when applied to diverse maturity profiles. The Khamis-Roche method uses the Fels Longitudinal Study to estimate adult height, classifying young individuals into groups based on predicted adult stature percentages (≥ 85.0% and < 90.0%, or ≥ 90.0% and < 95.0%) [6, 40–42]. These groupings, which cover the adolescent growth spurt, can be adjusted as needed using benchmarks for adult height at 18 years of age [6, 41, 42]. Therefore, the Khamis-Roche method is effective for assessing somatic maturation, with an error margin of approximately 2% in anthropometric measurements, which is consistent with previous research [42, 43] and should be used to monitor maturity consistently.

Metrics of match workload derived from Global Positioning System (GPS) technology, including total distance, high-speed running, sprint distance, and acceleration/deceleration actions, represent a comprehensive expression of players’ physical, technical, and tactical capacities during competition. Given that many of these capacities are significantly influenced by biological maturation, it is plausible that maturity-related differences extend beyond laboratory-based physical testing and manifest in competitive-matching requirements. Nevertheless, the extent to which biological maturation contributes independently to match workload variability beyond chronological age remains unclear in elite young soccer players.

Recent research suggests that biological maturity influences physiological and time-motion responses during small-sided games in adolescent male players. This finding underscores the potential impact of maturation on external load demands in soccer [44]. While the impact of biological maturation on physical performance is well documented, there is a paucity of evidence concerning the influence of maturation status on competitive match workload during adolescence in elite academy environments. Previous studies have predominantly employed cross-sectional designs, limited age ranges, or classifications based on maturity offset, which may not adequately reflect the complex interplay between chronological age and biological development. Moreover, few investigations have utilized longitudinal methodologies incorporating the PPAH to classify maturation status while modeling age × maturity interactions to match workload metrics. Owing to the limited representation of players in the earliest stages of maturation within elite academy environments, the pre- and circa-PHV categories were amalgamated to ensure sufficient analytical robustness. Although this strategy may diminish the sensitivity required to detect subtle differences between early maturation stages, chronological age was modeled as a continuous variable to maintain developmental resolution throughout adolescence. Consequently, this study sought to investigate the independent and interactive effects of chronological age and biological maturation on match workload parameters in elite youth soccer players over two competitive seasons. We hypothesized that (1) biological maturation status, as classified by PPAH, would exert a significant influence on match workload parameters, and (2) chronological age and biological maturation would interact to shape match workload demands, with more pronounced maturity-related effects evident at younger ages than at older ages.

## MATERIALS AND METHODS

### Experimental Approach to the Problem

This study used a longitudinal approach involving repeated measurements of the same participants. Sixty male soccer players were recruited from a professional soccer academy in Portugal, and various aspects of growth, maturation, and external workload were investigated. Biological maturity and workload assessment were conducted during the initial evaluation of the 2021–22 season, starting in the first week of July 2021 and concluding in the first week of May 2023 (Table 1). The research plan involved six assessment points spanning the 2021–22 and 2022–23 sports seasons, with three evaluations carried out per season, each conducted three months apart (Table 1). Players were divided into two categories according to their biological maturation level, as determined by the Khamis-Roche method [42]. The first category, Group 1, included participants in the pre- and circa-PHV stages (< 95.00% PAH), whereas the second category, Group 2, consisted of those in the post-PHV stage (≥ 95.00% PAH). Owing to the small number of athletes in the pre-PHV stage (n = 4), the analysis combined the pre-PHV and PHV classification groups. In the mixed-effects modeling framework, maturity status was treated as a time-varying variable, enabling players to transition between maturity groups at different assessment points as their percentage of predicted adult height increased. This methodology accurately captures the dynamic nature of biological development and reduces the potential classification bias over time. This study was correlational and could not determine causality. However, it identified associations between biological maturation and external workload parameters without inferring direct cause-andeffect relationships. The present study did not involve or assess any training intervention. These suggestions are hypothetical and based on the observed associations.

**TABLE 1:** Assessment during the two sports seasons.

Season	2021–22			2022–23		
Year	**2021**	**2022**	**2022**	**2022**	**2023**	**2023**
Month	August	January	May	August	January	May
Week	1^st^	1^st^	1^st^	1^st^	1^st^	1^st^
Assessment Number	1^st^	2^nd^	3^rd^	4^th^	5^th^	6^th^

### Subjects

Sixty male soccer players aged 12.0 to 18.1 years (mean 15.1 ± 1.1 years), with an average height of 174.9 cm (± 8.0 cm) and body mass of 64.1 kg (± 8.5 kg). Players belonging to the U14, U15, U16, and U17 age groups underwent their initial assessment for the 2020–21 season. All players completed the study without dropout or missing data. The inclusion criteria required participants to have at least three years of soccer experience, two seasons with the current academy/club, and no use of supplements that could affect growth and maturation. Players injured during the off-season and those who did not participate in the study were excluded. The players had an average of 5.5 ± 1.1 years of training/match experience at the national and international levels. This study was approved by the Faculty of Physical Education and Sports Ethics Committee of the University Lusófona. All participants were briefed on the procedures, and their parents or legal guardians signed an open-ended consent form allowing them to withdraw at any time. This study adhered to the ethical standards of the Declaration of Helsinki for human subject studies.

Given the longitudinal design and the use of mixed-effects modelling, statistical power was determined by both the number of participants (level 2 units) and the total number of repeated observations (level 1 units). The final dataset comprised 60 players assessed at six time points, resulting in approximately 360 observations. In multilevel analyses, statistical power is influenced by the number of clusters, cluster size, and intra-class correlation, with approximately 50 or more level-2 units generally considered sufficient for the stable estimation of fixed effects [45]. Considering the number of participants and repeated observations, the present study was adequately powered to detect small-to-moderate main and interaction effects within the mixed-effects framework.

### Procedures

#### Anthropometry and Body Composition

Anthropometric assessments were conducted in accordance with the standardized protocols of the International Society for the Advancement of Kinanthropometry (ISAK) [46]. All evaluations were performed by a certified and experienced ISAK Level I technician who ensured adherence to standardized measurement procedures. All measurements were obtained in the morning under controlled conditions, following an overnight fast, and at least 24 h without training to minimize acute physiological influences. The anthropometric variables included body mass, standing height, sitting height, and eight skinfold thicknesses (triceps, subscapular, biceps, suprailiac, abdominal, supraspinal, thigh, and calf). Standing and sitting heights were measured using an anthropometer (Seca model 206, Seca^®^, Hamburg, Germany) with an accuracy of 0.1 cm. Body mass was assessed using a calibrated electronic scale (Tanita model BC-601; Tanita Corporation, Tokyo, Japan) with an accuracy of 0.1 kg. The body mass index (BMI) was calculated as body mass divided by height squared (kg/m^2^). Skinfold thickness was measured using a Harpenden skinfold caliper (British Indicators Ltd., London, UK) with a precision of 0.1 mm. All anthropometric measurements were obtained in duplicate, and the mean of the two measurements was used for subsequent analysis. The intra-observer technical error of measurement (TEM) was maintained within the acceptable ISAK thresholds (< 1% for stature and body mass; < 5% for skinfold thickness). When the difference between the repeated measurements exceeded these limits, a third measurement was performed, and the median value was retained. The use of standardized ISAK procedures, duplicate measurements, and pre-defined error thresholds ensured high intra-observer reliability and measurement consistency.

#### Microcycle Contextualization

The weekly training regimen during the competition season included a match day (MD), followed by a rest day (MD+1/-6), and a recovery session (MD+2/-5). Pre-match training was organized as follows: MD+3/-4 focused on position-specific drills and small group games. MD+4/-3 emphasized tactical preparation for the upcoming match, featuring moderate-intensity positional exercises. MD+5/-2 involved activation drills that simulated certain technical and tactical aspects, ending with set-piece practice. MD+6/-1 was designated as the rest of the day for all the teams. The weekly schedule can be adjusted based on match-day developments. Young players engaged in four 90-minute weekly training sessions, with matches typically scheduled on weekends. The U14-U15 matches lasted 80 minutes, while the U16-U17 matches lasted 90 minutes, both played on outdoor artificial turf fields with 11-player teams. Academy players averaged 12 hours of combined weekly training, comprising four to five soccer sessions, one to two strength and power workouts, one to two speed and change of direction drills, and one competitive match per week.

#### Players Position

Sixty young male soccer players were categorized into seven distinct positions: goalkeepers (n = 7), central center-back players (n = 9), winger-back players (n = 10), central midfielders (n = 15), wingers (n = 12), and forward players (n = 7). While it is acknowledged that young soccer players (i.e., U-12 to U-18) may adopt various positions during training [47], the criterion for determining each player’s position was the most significant volume of play in a specific role.

#### Biological Maturation

Height, body mass, chronological age, and average parent height were used to predict the adult height of each player and to determine biological maturity [42] (Table 2). The heights of the biological parents of each player were self-reported and adjusted for overestimation using the equations proposed by Epstein, Valenki, Kalarchian, and McCurley (1995) [48]. Using the Khamis-Roche method, it is possible to predict the final adult height of men between 4.0 and 17.5 years old, considering a median error associated with the use of the 2.2 cm method, which is an average error that can vary between 0.8 and 2.8 cm [42]. As previously mentioned, players were classified into three groups based on their predicted adult height percentage (PPAH). Circa-PHVs accounted for 88–95% of the expected adult stature. Pre-PHVs were less than 88%, whereas post-PHVs exceeded 95% [41, 49, 50]. Two groups were established for data analysis based on biological maturation (maturity status). Group 1 comprised young soccer players with a predicted adult height of < 95%. Group 2 included players with a predicted adult height of ≥ 95%. The composition of each group was adjusted throughout the study period. Group 1 had 28 players at the initial evaluation, whereas Group 2 had 32. The distribution of samples between the two groups (pre- and circa-PHV combined vs. post-PHV) was also evaluated. Given the limited number of players classified as pre-PHV (n = 4), the pre- and circa-PHV categories were combined (< 95% PAH) to ensure an adequate group size and statistical robustness within the mixed-effects models. Multilevel analyses require sufficient subgroup sizes for the reliable estimation of fixed and interaction effects, as very small subgroups may lead to unstable parameter estimates and inflated standard error. Furthermore, the inclusion of chronological age as a continuous covariate in the models allowed for the identification of developmental trends across adolescence, partially mitigating the potential heterogeneity within the combined group.

**TABLE 2:** Anthropometric, Body Composition, Maturity characteristics and Match running metrics from under-14 to under-17.

Maturity Group	Pre/Circa-PHV (n = 28)	Post-PHV (n = 32)
**Anthropometric and maturity characteristics**		
Chronological Age (years)	13.8 ± 0.6	15.4 ± 0.8
Height (cm)	166.8 ± 5.3	175.6 ± 6.5
Body Mass (kg)	53.4 ± 5.5	65.4 ± 6.3
PPAH (%)	92.3 ± 1.9	97.8 ± 1.5

**Match running metrics**		
Total distance (m/min)	137.1 ± 16.5	141.7 ± 16.6
High-speed running (m/min)	1.5 ± 0.7	1.33 ± 0.7
Sprint distance (m/min)	0.01 ± 0.0	0.01 ± 0.0
Total Accelerations/Decelerations (cnt/min)	0.02 ± 0.0	0.02 ± 0.0

Key: PHV = Peak Height Velocity; PPAH = Percentage of Predicted Adult Height.

#### External Workload

Throughout the 2021–22 and 2022–23 seasons, each player participating in official competitive league matches was equipped with a personal 10-Hz GPS unit (APEX, STATSports, UK) incorporating a 100-Hz triaxial accelerometer. The 10-Hz GPS technology has previously demonstrated acceptable validity and reliability for quantifying total distance, high-speed running, sprint performance, and acceleration/deceleration demands in team sports contexts [51]. All matches were conducted in accordance with national federation regulations for each age category and were played on full-sized pitches compliant with official competition standards. The analysis was restricted exclusively to official competitive matches. GPS devices were positioned on the upper back of each participant using a flexible neoprene vest to ensure consistent placement across matches. Following each match, data were downloaded and analyzed using the STATSports software package (Sonra 3.0, 2020). The present study focused on five match-running metrics, as detailed in Table 3. Absolute speed thresholds were applied uniformly across all players. To ensure meaningful workload exposure, only players who completed a minimum of 60 minutes of match play were included in the analysis, consistent with previous GPS-based investigations in elite youth soccer. To facilitate standardized comparisons across matches and players, all workload metrics were normalized relative to individual playing time. Specifically, values were divided by total playing time and multiplied by 60 to express outcomes on a per-60-minute basis. This approach enables equitable comparisons while preserving data integrity.

**TABLE 3:** Definition of GPS metrics used for monitoring match external workload from under-14 to under-17.

GPS Metric	Definition
Total distance (m/min)	The total distance covered at all speeds per minute
High speed running distance (m/min)	The distance covered at ≥ 5.5–6.99 m · s^−1^ per minute
Sprint running distance (m/min)	The distance covered ≥ 7.0 m · s^−1^ per minute
Accelerations (n/min)	The number of accelerations above 3.0 m · s^−2^
Decelerations (n/min)	The number of decelerations above 3.0 m · s^−2^
Total of Accelerations and Decelerations (n/min)	The sum of the total number of accelerations and of decelerations above 3.0 m · s^−2^

### Statistical Analysis

Zero-order Pearson correlations were first performed to examine the potential overlap among the performance metrics, with correlations ranging from 0.02 to 0.61, indicating low-to-moderate associations between the variables (Table 4). These findings suggest that each parameter retained sufficient independence for subsequent modeling. Descriptive statistics are presented as mean ± standard deviation (SD). To investigate changes in external workload parameters (total distance per minute, high-speed running per minute, sprint distance per minute, and total accelerations and decelerations per minute) as a function of chronological age and maturity status (pre- and circa-PHV players coded as 0; post-PHV players coded as 1), twolevel linear growth models were used. At Level 1, repeated measurements over time were modeled to capture the within-player changes. For graphical presentation and improved interpretability, the outcome variables were mean-centered; however, chronological age was entered in its original scale (years) and was not standardized. Chronological age was entered as a continuous predictor, and an interaction term (age × maturity status) was computed to examine the differential developmental trajectories between maturity groups. At Level 2, between-player differences in growth trajectories were examined according to maturity status. Multilevel linear mixed models were constructed for each dependent variable: (1) age as the sole fixed effect, (2) age and maturity status as fixed effects, and (3) age and maturity status and their interaction as fixed effects. Playing position was included as a covariate to control for positional demands. Player ID was included as a random intercept in all models to account for repeated measurements and nested data structures. Residual normality was assessed using histograms and Q–Q plots, and homoscedasticity was examined using the residual plots. Variance Inflation Factors (VIFs) were calculated to check for multicollinearity among predictors, and all values were below the conventional threshold of 5, indicating no collinearity concerns. Model fit was compared using log-likelihood values and the Akaike Information Criterion (AIC). In addition to reporting unstandardized β coefficients and 95% confidence intervals, standardized effect size estimates were calculated to enhance the interpretability and practical relevance of significant findings. All statistical analyses were performed using SPSS version 28.0 (IBM Corp., Armonk, NY, USA).

**TABLE 4:** Pearson correlation coefficients (r), p-values, and 95% confidence intervals among external load variables expressed per minute.

Variable 1	Variable 2	r	p-value	CI 95%
Total Distance	High Speed Running	0.318	0.001	[0.275, 0.359]
Total Distance	Sprint Distance	0.022	0.061	[-0.002, 0.091]
Total Distance	Total Accelerations and Decelerations	-0.114	0.001	[-0.160, -0.068]
High Speed Running	Sprint Distance	0.611	0.001	[0.589, 0.647]
High Speed Running	Total Accelerations and Decelerations	0.193	0.001	[0.148, 0.237]
Sprint Distance	Total Accelerations and Decelerations	0.243	0.001	[0.199, 0.287]

## RESULTS

**FIG. 1:**
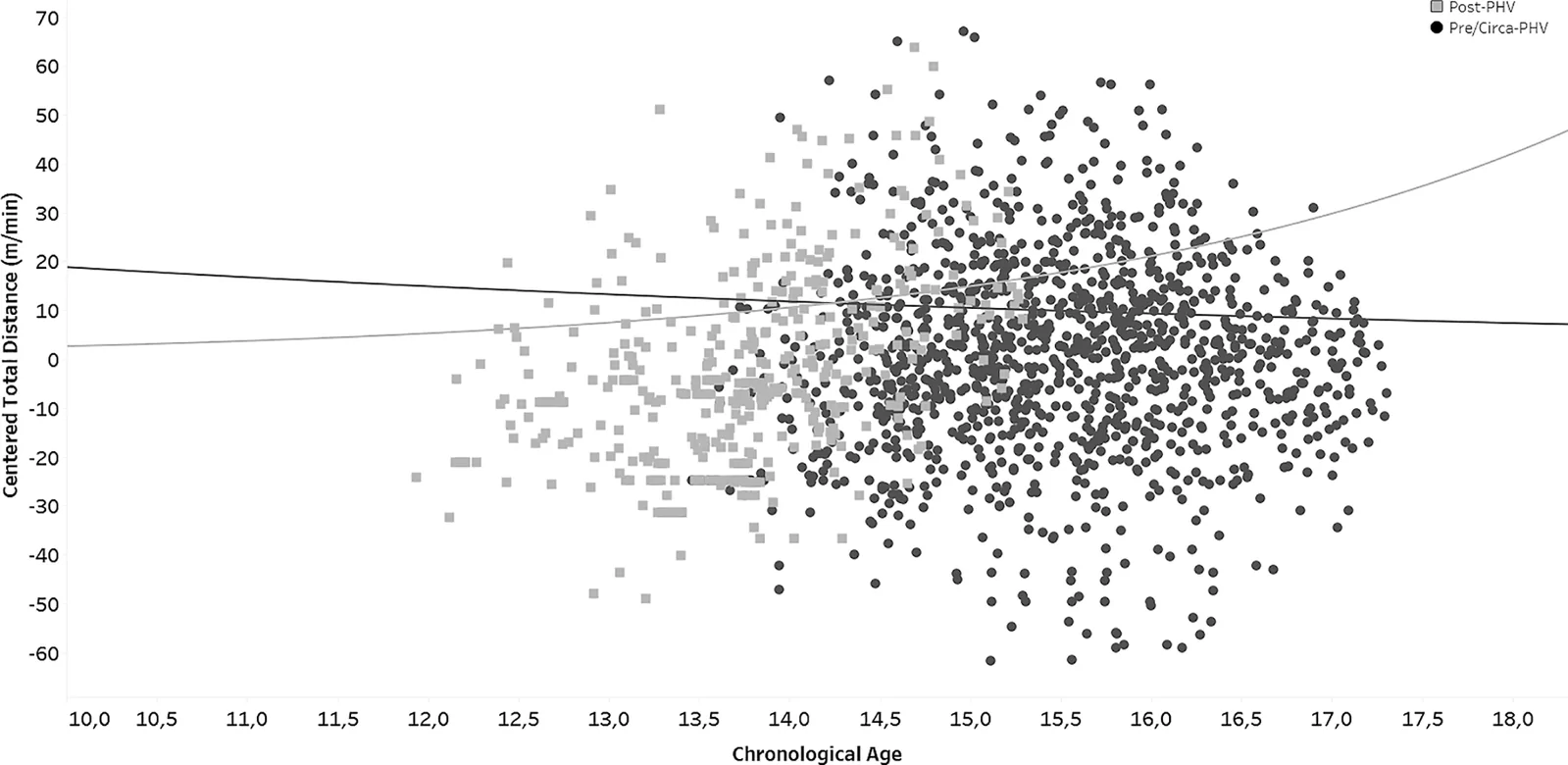
Representation of the Total Distance (m/min) in terms of chronological age evolution according to maturity status. Note: Key: Pre-Peak height velocity; Circa-PHV = Circa-Peak height velocity; Post-PHV = Post-Peak height velocity.

For total distance per minute (TD/min), Model 1 revealed a significant effect of age (β = -2.371, p = 0.001). Model 3, which incorporated maturity status and the age × maturity interaction, provided the best fit (AIC = 14481.497). The ΔAIC values relative to Models 1 and 2 exceeded the conventional threshold of 2. Model 3 demonstrated significant effects for the intercept (β = -207.282, p = 0.001), age (β = 14.828, p = 0.001), maturity status (β = 133.276, p = 0.001), and interaction term (β = -9.378, p = 0.001) (Table 5; Figure 1).

**TABLE 5:** Mixed-Effects Model Results for All Metrics.

Metric	Model	Intercept (p-value)	Age (p-value)	Maturity (p-value)	Interaction (p-value)	AIC	Random Effect Variance
Total distance/min	1	34.838 (0.002)	-2.371 (0.001)	–	–	14513.913	145.546
	2	34.497 (0.003)	-2.331 (0.007)	-0.145 (0.929)	–	14511.076	145.648
	3	-207.282 (0.001)	14.828 (0.001)	133.276 (0.001)	-9.378 (0.001)	14481.497	133.987

High-speed running/min	1	4.414 (0.001)	0.527 (0.001)	–	–	7536.351	5.250
	2	-8.628 (0.001)	0.614 (0.001)	-0.323 (0.001)	–	7535.250	5.228
	3	-0.032 (0.996)	0.005 (0.991)	-5.073 (0.128)	0.333 (0.153)	7534.281	5.194

Sprint distance/min	1	-2.627 (0.001)	0.174 (0.001)	–	–	3982.742	0.516
	2	-2.675 (0.001)	0.179 (0.001)	-0.020 (0.001)	–	3986.050	0.516
	3	-0.638 (0.769)	0.035 (0.820)	-1.146 (0.326)	0.079 (0.334)	3988.291	0.513

Total accelerations and decelerations/min	1	-0.905 (0.001)	0.060 (0.001)	–	–	1532.902	0.099
	2	-1.276 (0.001)	0.103 (0.001)	-0.158 (0.001)	–	1517.752	0.097
	3	-4.746 (0.001)	0.349 (0.001)	1.761 (0.002)	-0.134 (0.001)	1510.667	0.097

**FIG. 2:**
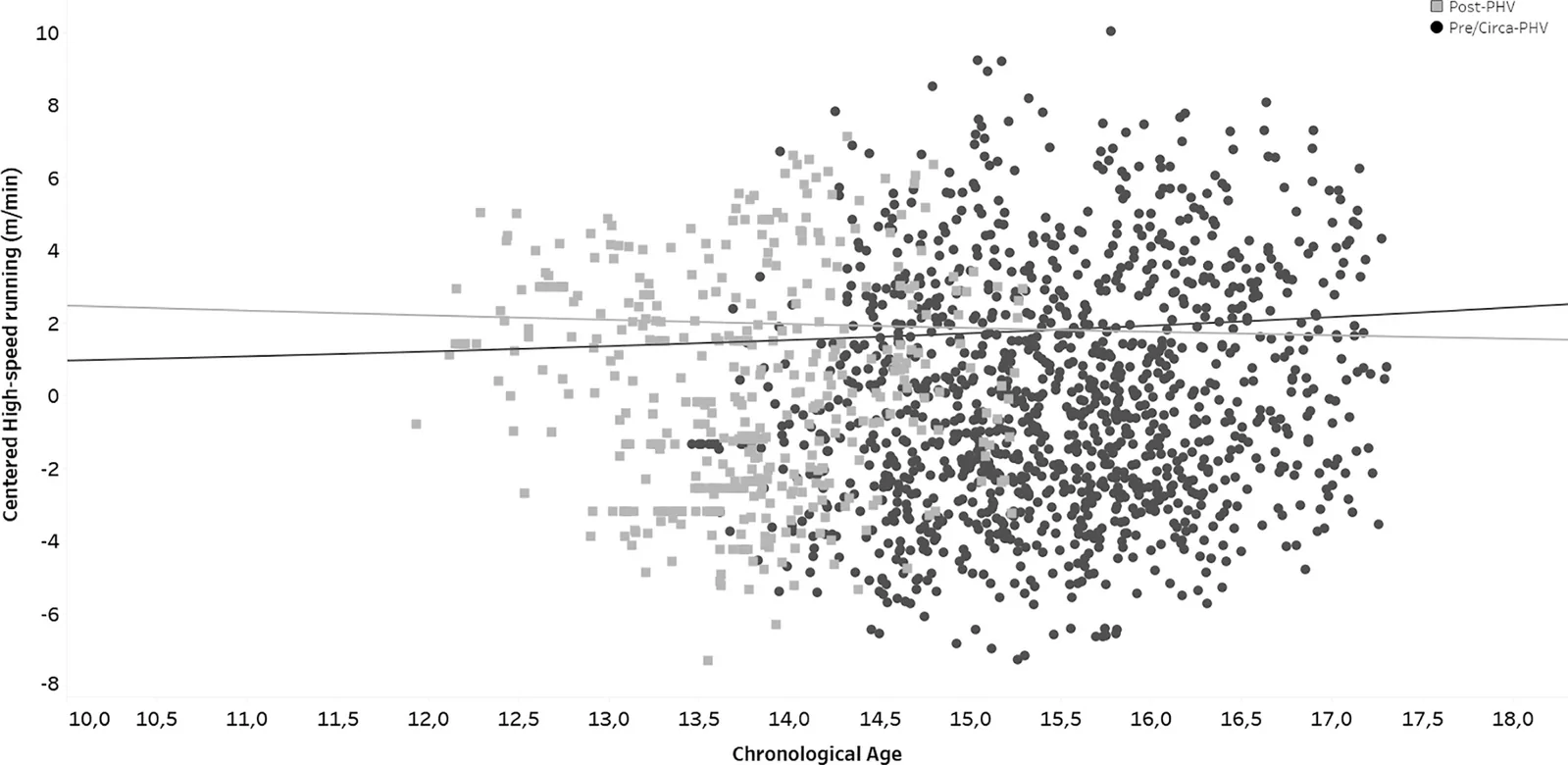
Representation of the High-Speed Running (m/min) in terms of chronological age evolution according to maturity status. Note: Key: Pre-Peak height velocity; Circa-PHV = Circa-Peak height velocity; Post-PHV = Post-Peak height velocity.

For high-speed running per minute (HSR/min), Model 1 showed a significant effect of age (β = 0.527, p = 0.001). Model 2 demonstrated significant effects of age (β = 0.614, p = 0.001) and maturity status (β = -0.323, p = 0.001). Model 3 yielded the lowest AIC (7534.281); however, no significant effects were observed for the intercept (β = -0.032, p = 0.996), age (β = 0.005, p = 0.991), maturity status (β = -5.073, p = 0.128), or the interaction term (β = 0.333, p = 0.153). ΔAIC comparisons did not indicate a meaningful improvement in model fit relative to simpler models; therefore, the interaction effect was not retained for interpretation (Figure 2).

**FIG. 3:**
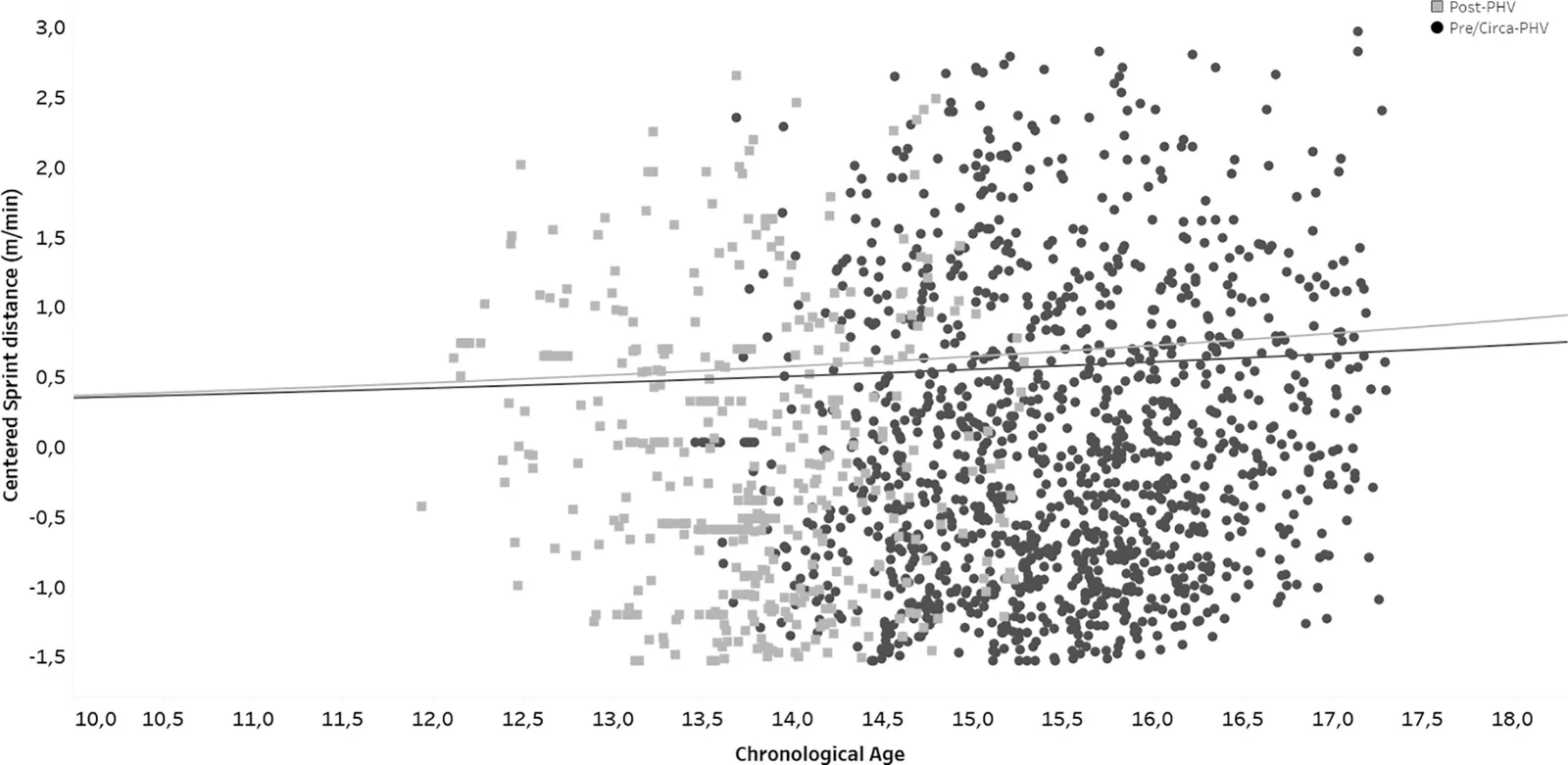
Representation of the Sprint Distance (m/min) in terms of chronological age evolution according to maturity status. Note: Key: Pre-Peak height velocity; Circa-PHV = Circa-Peak height velocity; Post-PHV = Post-Peak height velocity.

For sprint distance per minute (SD/min), Model 1 revealed a significant effect of age (β = 0.174, p = 0.001). Model 2 showed significant effects of age (β = 0.179, p = 0.001) and maturity status (β = -0.020, p = 0.001). In Model 3, no significant effects were observed for the intercept (β = -0.638, p = 0.769), age (β = 0.035, p = 0.820), maturity status (β = -1.146, p = 0.326), or interaction term (β = 0.079, p = 0.334). ΔAIC comparisons did not support a meaningful improvement in model fit relative to simpler models; therefore, the interaction effect was not retained for interpretation (Figure 3).

**FIG. 4:**
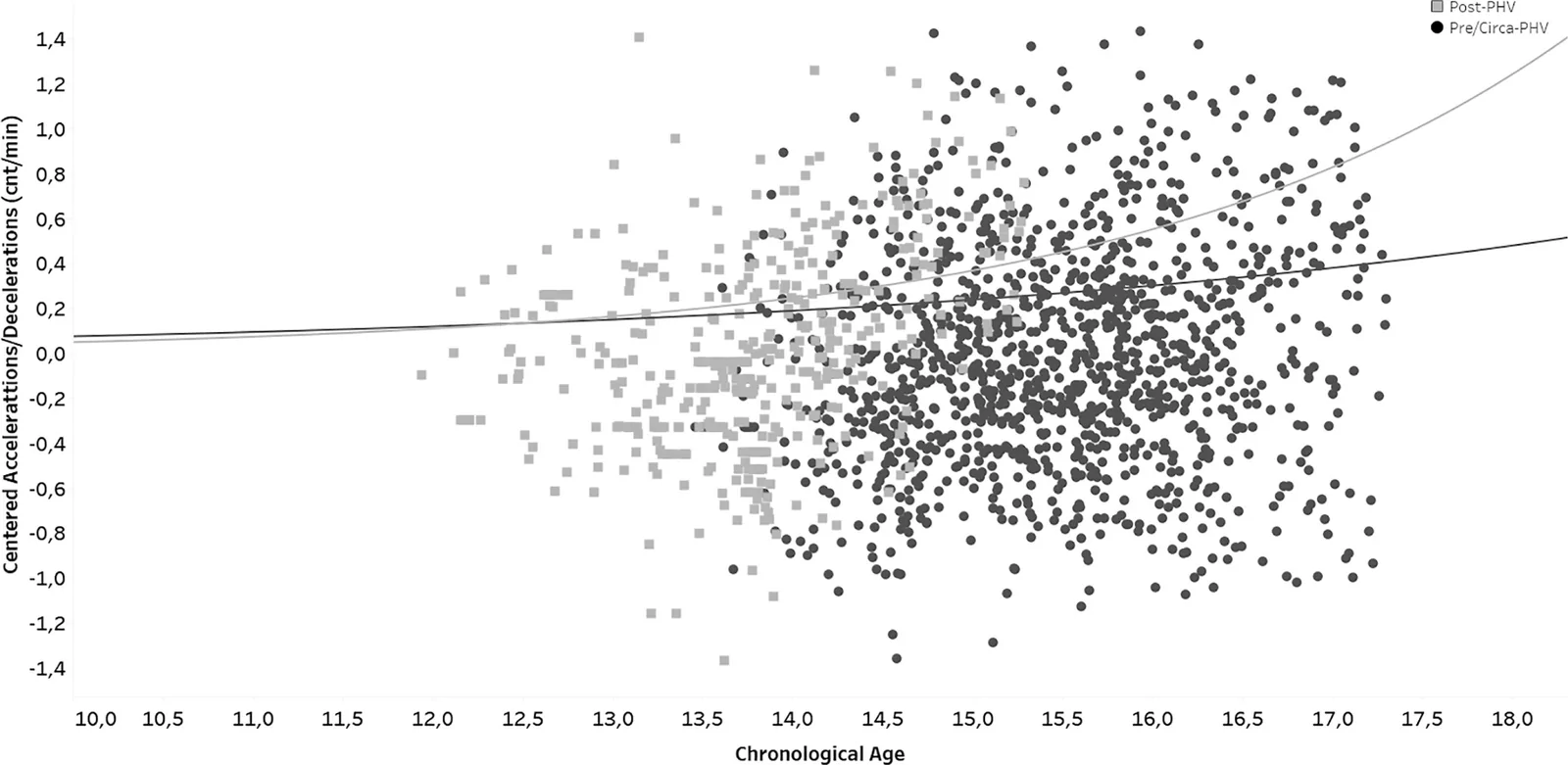
Representation of the Accelerations/ Decelerations (cnt/min) in terms of chronological age evolution according to maturity status. Note: Key: Pre-Peak height velocity; Circa-PHV = Circa-Peak height velocity; Post-PHV = Post-Peak height velocity.

For total accelerations and decelerations per minute (Total Ac & Dec/min), Model 3 provided the best fit (AIC = 1510.667), with ΔAIC values exceeding the threshold of 2, relative to Models 1 and 2. Model 3 demonstrated significant effects for the intercept (β = -4.746, p = 0.001), age (β = 0.349, p = 0.001), maturity status (β = 1.761, p = 0.002), and interaction term (β = -0.134, p = 0.001) (Figure 4).

## DISCUSSION

The primary aim of this study was to evaluate the impact of maturity status on match workload parameters over two consecutive seasons and explore the interaction between these effects and chronological age. The principal findings were as follows: (1) the effect of maturity on TD/min diminished with advancing age; (2) chronological age had a significant impact on HSR/min and SD/min, whereas maturity exhibited a less consistent influence on these parameters; and (3) for Total Ac & Dec/min, both age and maturity were significant predictors, although the influence of maturity decreased throughout adolescence.

In alignment with the existing literature, chronological age was found to be significantly correlated with TD/min, HSR/min, and SD/min. Previous studies have indicated that older youth players typically exhibit enhanced high-intensity running capacity and distinct match-running profiles compared with their younger counterparts [28, 29, 52, 53]. However, unlike earlier cross-sectional studies, the current research employed a longitudinal approach to model age × maturity interactions, thereby offering additional insights into the temporal evolution of maturation-related effects.

In the context of TD/min, Model 3 revealed a significant interaction between age and maturity status. These results are consistent with those of Figueiredo et al. [15], who observed that physical development has a more pronounced impact on performance during early adolescence. Similarly, Parr et al. [53] emphasized the influence of maturation on match performance among youth players. The current findings build upon this body of work by illustrating that maturity-related differences in TD/min diminish with age when examined within a multilevel framework across two competitive seasons. As maturation advances and anthropometric characteristics become more uniform, the relative impact of early physical development may decrease, although the underlying mechanisms were not directly assessed in the present study [15].

In relation to HSR/min, age was identified as the primary determinant, aligning with the findings of Buchheit and Mendez-Villanueva [17], who noted that early maturing players tend to excel in high-speed tasks. However, the lack of significant interaction effects in the current study indicates that the impact of biological maturity on HSR becomes less distinct as players progress through adolescence and approach adulthood. Lovell et al. [54] similarly found that maturation-related advantages in high-intensity activities diminish with age. Therefore, while HSR/min increases with chronological age, its association with maturity status appears less pronounced when age is controlled. While this pattern may reflect broader developmental changes during adolescence, such as improvements in neuromuscular or tactical characteristics, these mechanisms were not directly evaluated in the present investigation and should therefore be interpreted cautiously.

A similar pattern was observed for the SD/min. Although age had a significant impact on sprint distance, the interaction between age and maturity was not significant. These findings are consistent with those of Lovell et al. [54], who noted that the effects of maturation diminish as technical and tactical factors gain prominence in later developmental stages. Collectively, these results may indicate that sprint-related metrics may be more closely linked to general age-related neuromuscular development than to maturity status during mid-to-late adolescence. However, this interpretation remains inferential, as technical and tactical variables were not directly measured.

In the analysis of Total Ac & Dec/min, both age and maturity, along with their interaction, were found to be significant factors. These findings align with previous research, suggesting that early maturers exhibit advantages in activities reliant on strength and power during youth [11, 40]. Figueiredo et al. [11] underscored the influence of biological maturation on explosive actions, while subsequent studies have highlighted that these advantages may diminish as players mature and their technical efficiency improves [40]. The current study builds on these findings by illustrating that the effects of maturation on multidirectional match demands are moderated by age across all seasons.

Recent research has notably expanded this perspective to include training contexts. Cherni et al. [44] demonstrated that biological maturity status significantly influences physiological and time-motion responses during small-sided games among adolescent soccer players, with running demands varying by maturity level and game format. When considered in conjunction with the present findings, this suggests that maturity-related differences in competitive match workload may also be evident during training sessions, particularly in formats that impose substantial external load demands on players. Consequently, practitioners should consider maturity status when interpreting match workload metrics and when designing and monitoring training tasks, such as small-sided games.

The longitudinal design and application of PPAH for classifying maturity status constitute a significant methodological advancement. Prior research has often depended on maturity offset methods or cross-sectional age group comparisons [28, 53]. By incorporating repeated observations within players and explicitly examining age × maturity interactions, this study offers a more comprehensive understanding of how biological maturation influences match workload during adolescence.

### Limitations and future directions

The relatively limited sample size may constrain the generalizability of the findings to broader populations and varying competition levels. Future research should incorporate more diverse cohorts to enhance the external validity of these results. Although this study focused on external load parameters, a more comprehensive approach that includes internal workload measures (e.g., heart rate responses, perceived exertion, or neuromuscular markers) could offer additional insights into the interaction between biological maturation and match demands. Although the present study employed a longitudinal design across two competitive seasons, the observational nature of the data precludes causal inference regarding the long-term impact of biological maturation on player development. Furthermore, defining and standardizing the methodologies used to assess biological maturation, as well as the metrics and speed thresholds employed to quantify external load, remain essential for improving comparability across studies. Another limitation pertains to the categorical classification of maturity status (pre-/circa-PHV vs. post-PHV), which may reduce the sensitivity in detecting subtle maturity-related differences and may partially limit model precision. Future studies should explore continuous indicators, such as the discrepancy between biological and chronological age (e.g., PPAH deviation), which may provide a more refined approach for identifying performance variations, particularly in heterogeneous age groups. Continuous maturity metrics may offer greater analytical resolution by capturing the gradual and dynamic nature of adolescent development and may enhance the modeling of interaction and nonlinear effects across developmental stages. Future research should investigate how biological maturation monitoring can be integrated into training load and injury prevention frameworks. Recent evidence suggests that combining PHV monitoring with workload metrics may enhance the identification of periods of increased injury susceptibility during adolescence [55]. Given that maturity-related effects on match workload were more pronounced at younger ages in the present study, integrating maturity assessment (e.g., PPAH or PHV tracking) into longitudinal load management strategies may support more individualized training prescriptions and potentially mitigate growth-related injury risks in elite youth academies. Future investigations should include samples representing players across all stages of biological maturation and extend analyses to female soccer players to determine whether similar maturity-related workload patterns are observed across sexes.

## CONCLUSIONS

This study demonstrates that the impact of biological maturation on match workload is age-dependent and variable-specific in elite youth soccer. While chronological age predominantly determines HSR/min and SD/min, biological maturation significantly interacts with age to influence TD/min and Total Ac & Dec/min. Notably, maturity-related effects are more pronounced in younger players and progressively attenuate during adolescence. These findings highlight the importance of integrating maturity status into the interpretation of match workload metrics rather than relying solely on chronological age as a determinant. Incorporating biological maturation into performance monitoring and talent development processes, particularly during early adolescence, may help prevent the overestimation of early maturing players and the underestimation of later-maturing counterparts.

### Practical Applications

The findings of this study have several implications for coaches and practitioners of young players. First, the significant influence of age on performance metrics suggests that training programs should be tailored to athletes’ developmental stages. Younger players may benefit from focusing on developing fundamental skills and physical attributes, whereas older players may incorporate more advanced training techniques and tactics. Second, this study highlights the importance of biological maturation in player selection and the development of skills. Early maturing players may possess physical advantages; however, ensuring that their development is balanced and sustainable is crucial. Coaches should monitor the progress of all players, regardless of their maturation status, and provide appropriate support and guidance. Finally, the findings emphasize the need for individualized training programs that account for the unique needs of each player. While age and maturity status can provide valuable insights, it is essential to consider other factors such as playing position, injury history, and individual preferences. By tailoring training programs according to the specific characteristics of each athlete, coaches can optimize their development and maximize their potential performance.

## Data Availability

The database supporting the conclusions of this article is available from the corresponding author on reasonable request.
